# Preschool Gender-Typed Play Behavior at Age 3.5 Years Predicts Physical Aggression at Age 13 Years

**DOI:** 10.1007/s10508-017-1005-6

**Published:** 2017-06-23

**Authors:** Karson T. F. Kung, Gu Li, Jean Golding, Melissa Hines

**Affiliations:** 1 Department of Psychology, Gender Development Research Centre, University of Cambridge, Cambridge, CB2 3RQ UK; 2 Centre for Child and Adolescent Health, School of Social and Community Medicine, University of Bristol, Bristol, UK

**Keywords:** Gender role, Play, Aggression, Adolescence, ALSPAC, Gender identity

## Abstract

Gender differences in play behavior and physical aggression have been consistently reported. Theoretical perspectives concerning evolutionary, social, and social-cognitive mechanisms suggest that male-typical play behavior during childhood increases subsequent physical aggression. The evidence supporting these connections is limited, however. The present study investigated the association between gender-typed play behavior in early childhood and physical aggression in early adolescence using a sample drawn from a longitudinal, population study, the Avon Longitudinal Study of Parents and Children. Based on gender-typed play behavior as measured by the Pre-School Activities Inventory at age 3.5 years, samples of masculine (64 boys, 60 girls), feminine (80 boys, 66 girls), and randomly selected control children (55 boys, 67 girls) were recruited at age 13 years and administered the Reinisch Aggression Inventory. After controlling for a range of sociodemographic variables, maternal characteristics, and behavioral problems, including hyperactivity and conduct problems at age 3.5, significant group differences in physical aggression at age 13 were found among children classified as masculine, control, and feminine at age 3.5. Masculine children exhibited significantly more physical aggression than control children or feminine children, and control children exhibited significantly more physical aggression than feminine children. The association between gender-typed play behavior and physical aggression was not moderated by sex. These results suggest that the degree of childhood gender-typed play behavior independently predicts the degree of physical aggression at adolescence in boys and in girls.

## Introduction

Gender differences in physical aggression have been well documented, with males typically exhibiting more physical aggression than females. Gender differences in physical aggression emerge in early childhood (Alink et al., 2006; Baillargeon et al., 2007) and are maintained throughout childhood and into adulthood (Archer, 2004; Card, Stucky, Sawalani, & Little, 2008; Hyde, 1984). Magnitudes, or effect sizes, for gender differences can be evaluated using Cohen’s *d* statistic. Values of *d* of 0.2, 0.5, and 0.8 are considered to be small, medium, and large, respectively (Cohen, 1988). In regard to gender differences in physical aggression in children and adolescents, a meta-analysis by Card et al. found that the weighted gender difference across studies of child and/or adolescent samples approached a large magnitude (*d* = 0.73). It is noteworthy that this weighted effect size is relatively large in the area of psychological research. Hyde (2005) reviewed 46 meta-analyses on gender differences in psychological traits and found that, among the 124 weighted effect sizes reported in these analyses, less than 10% were larger than *d* = 0.65.

Another area of behavior that shows large gender differences is childhood play behavior. Starting from early childhood, boys prefer toy guns, swords, and vehicles, whereas girls prefer dolls, tea sets, and other domestic toys (Alexander, Wilcox, & Woods, 2009; O’Brien & Huston, 1985; Pasterski et al., 2005; Ruble, Martin, & Berenbaum, 2006; Servin, Bohlin, & Berlin, 1999; Sutton-Smith & Rosenberg, 1971). Boys and girls also engage in sex-segregated play, spending more time with playmates of their own, than the other, sex (Howes, 1988; LaFreniere, Strayor, & Gauthier, 1984; Maccoby & Jacklin, 1987; Pellegrini, Long, Roseth, Bohn, & van Ryzin, 2007). There are also gender differences in play styles, with boys engaging in more rough-and-tumble play with peers and fathers than do girls (Jacklin, DiPietro, & Maccoby, 1984; Maccoby, 1998; Maccoby & Jacklin, 1987; Pitcher & Shultz, 1983). Notably, gender differences in childhood play behavior can be very large. For example, studies involving thousands of preschoolers suggest that the magnitude of gender differences in gender-typed play behavior assessed by a parent-report questionnaire, the Pre-School Activities Inventory (PSAI), is greater than *d* = 2.0 (Golombok & Rust, 1993a, 1993b; Golombok et al., 2008; Iervolino, Hines, Golombok, Rust, & Plomin, 2005).

Various theoretical perspectives suggest that male-typical play behavior during childhood increases subsequent physical aggression. Key theoretical perspectives concern evolutionary, social, and social-cognitive mechanisms. Evolutionary theorists suggest that rough-and-tumble play allows children, especially boys, to learn fighting techniques that may be valuable later in life (Archer, 1994; Pereira & Altmann, 1985). The social learning perspective suggests that, because boys’ play is more active, physical, and competitive, having boys as playmates may create more opportunities for physical contact and thus facilitate and reinforce the expression of physical aggression (Côté, 2007; Maccoby, 1998). It has also been suggested that, via social-cognitive mechanisms such as associative priming, the presence of male-typical and aggressive toys, such as guns, swords, and action figures, may elicit aggressive behavior during play and gradually produce a desensitization to violence (Berkowitz, 1974, 1984; Cline, Croft, & Courrier, 1973).

Previous research has provided some support for the hypothesized positive association. While there is generally a lack of an association between child-to-child rough-and-tumble play and physical aggression (Pellegrini, 1988, 2006), more frequent rough-and-tumble play with the father during childhood has been related to increased physical aggression in retrospective (Paquette, Carbonneau, Dubeau, Bigras, & Tremblay, 2003), contemporaneous (Flanders, Leo, Paquette, Pihl, & Séguin, 2009), and longitudinal (Flanders et al., 2010) research. In addition, one study found that children direct more physical aggression toward male than female peers during play (Ostrov & Keating, 2004). Thus, sex-segregated play could lead to greater physical aggression in boys than in girls. Furthermore, although a few studies have reported no significant association between playing with male-typical and aggressive toys, such as toy guns and swords, and aggression (Etaugh & Happach, 1979; Sutton-Smith, Gerstmyer, & Meckley, 1988), other studies have found increased physical aggression in children playing with such toys during play sessions (Feshbach, 1956; Hellendoorn & Harinck, 1997; Potts, Huston, & Wright, 1986; Sanson & Di Muccio, 1993; Turner & Goldsmith, 1976; Watson & Peng, 1992). It has also been reported that playing with such toys increases physical aggression immediately after play sessions (Feshbach, 1956; Turner & Goldsmith, 1976). Hence, there is some evidence suggesting that male-typical play behavior may increase physical aggression during childhood.

Nonetheless, the aforementioned studies relating childhood gender-typed play behavior and physical aggression contain certain limitations. Firstly, some of the studies either included only boys or found an association only in boys, perhaps due to low prevalence of male-typical play behavior in girls. Secondly, many of the studies looked at concurrent associations in preschoolers and school-aged children, providing little information on the longer-term effects of childhood gender-typed play behavior on physical aggression. The few studies that went beyond concurrent associations focused on immediate effects or subsequent aggressive behavior during childhood, so that little is known about whether the effects may extend into adolescence. Thirdly, prior studies tended to employ measures of play with unknown psychometric properties. These limitations argue for additional longitudinal research examining whether childhood gender-typed play behavior predicts physical aggression in adolescent boys and girls using a psychometrically constructed assessment of childhood gender-typed play behavior.

The present study was designed to investigate whether gender-typed play behavior in early childhood predicts physical aggression in early adolescence, and recruited participants from the Avon Longitudinal Study of Parents and Children (ALSPAC). ALSPAC is a longitudinal population study in the UK that included the PSAI to measure childhood gender-typed play behavior. At age 13 years, subsamples of extremely masculine boys and girls, extremely feminine boys and girls, and randomly selected boys and girls, categorized according to their PSAI score at age 3.5 years, were selected. At age 13, the selected individuals were administered the Reinisch Aggression Inventory (RAI; Reinisch & Sanders, 1986). The RAI measures physical aggression, as well as verbal aggression, non-aggressive coping, and withdrawal, in situations involving interpersonal conflicts. It was hypothesized that children who were more masculine at age 3.5 would show higher levels of physical aggression at age 13. Relationships of childhood gender-typed play behavior to verbal aggression, non-aggressive coping, and withdrawal were also examined.

## Method

### Participants and Procedure

The children were recruited from ALSPAC, a longitudinal population study of over 14,000 mothers and their children beginning prenatally (Boyd et al., 2013). The ALSPAC cohort consists of pregnant women who were residents within Avon, a geographically defined area in Southwest England, and had expected delivery dates between April 1, 1991, and December 31, 1992 (Boyd et al., 2013). The current study followed up a subsample of the cohort who were selected based on childhood gender-typed play behavior.

At 3.5 years of age, gender-typed play behavior was assessed using the PSAI, a psychometrically constructed parent-report questionnaire specifically designed to differentiate gender-typed play behavior within, as well as between, the sexes. Using PSAI scores at age 3.5 years, six groups of children were selected for follow-up: boys (*n* = 122) and girls (*n* = 111) with extremely masculine scores (masculine children), boys (*n* = 110) and girls (*n* = 109) with extremely feminine scores (feminine children), and boys (*n* = 99) and girls (*n* = 108) randomly selected from among the remaining children (control children). Among these children, 64 masculine boys and 60 masculine girls, 80 feminine boys and 66 feminine girls, and 55 control boys and 67 control girls participated at age 13 years and completed the aggression measure, representing 59% of the age 3.5 years sample. This participation rate was slightly higher than the 54% of adolescents from the entire ALSPAC sample who continued to take part in other aspects of the study at age 13 years. There was no significant difference between boys and girls in the distribution of membership across the three childhood gender-typed play behavior groups at either time point. Scores of all feminine and masculine children were at least 1 *SD* from the standardized norms for their own sex (boys: *M* = 60, *SD* = 10; girls: *M* = 40, *SD* = 10), and mean PSAI scores for the control children were close to means of the standardized norms for their own sex. In addition, there was no significant difference in mean PSAI scores in the six groups between the cohort selected at age 3.5 years and the subsample still participating at age 13 years. Descriptive statistics of PSAI scores for the six groups at age 3.5 and 13 years are shown in Table 1. There was no differential withdrawal among groups of girls, *χ* ^*2*^(2, *n* = 328) = 1.63, *p* = .44, or between masculine and control boy groups, *χ* ^*2*^(1, *n* = 221) = 0.11, *p* = .74. However, feminine boys were more likely to participate at age 13 years than masculine boys, *χ* ^*2*^(1, *n* = 232) = 9.34, *p* = .002, or control boys, *χ* ^*2*^(1, *n* = 209) = 6.72, *p* = .01.

**Table 1:** Descriptive statistics of Pre-School Activities Inventory (PSAI) scores within each childhood gender-typed play behavior group in original and follow-up samples

	Boys				Girls			
	*N*	*M*	*SD*	Range	*N*	*M*	*SD*	Range
Original age 3.5 sample								
Masculine children	122	80.68	4.07	75.75–95.55	111	56.91	4.58	51.55–71.35
Control children	99	62.02	6.13	49.35–74.65	108	34.84	7.51	22.95–49.35
Feminine children	110	43.96	4.61	20.75–48.25	109	17.10	4.16	4.25–21.85
Follow-up age 13 sample								
Masculine children	64	80.77	4.64	75.75–95.55	60	56.78	4.80	51.55–70.25
Control children	55	61.33	5.87	49.35–74.65	67	35.31	7.72	22.95–49.35
Feminine children	80	44.02	4.49	20.75–48.25	66	17.15	4.16	5.35–21.85

Higher PSAI scores indicate more masculine behavior. The standardized norm is *M* = 60, *SD* = 10 for boys and *M* = 40, *SD* = 10 for girls (Golombok & Rust, 1993a, 1993b)

The age 13 sample was representative of the geographic area of Avon, England, and diverse in socioeconomic background. Based on paternal occupation, about 50% of the sample was professional/managerial/technical and 50% skilled/partly skilled/unskilled. Regarding ethnicity, 96% of the children were of Caucasian descent.

Ethical approval for the study was obtained from the ALSPAC Ethics and Law Committee and the local research ethics committees. Please note that the study website contains details of all the data that are available through a fully searchable data dictionary (http://www.bris.ac.uk/alspac/researchers/data-access/data-dictionary/).

### Measures

#### Gender-Typed Play Behavior at Age 3.5 Years

The Pre-School Activities Inventory (PSAI; Golombok & Rust, 1993a, 1993b) is a 24-item parent-report questionnaire answered using a 5-point Likert scale. The questions address three aspects of gender-typed behavior: gender-typed toy preferences (e.g., dolls, guns, swords), gender-typed activities (e.g., playing at having a male occupation, playing with girls), and gender-typed child characteristics (e.g., enjoys rough-and-tumble play, likes pretty things). A full list of items can be found in the standardization study (Golombok & Rust, 1993a). The PSAI is psychometrically constructed to measure gender-typed play behavior as a single trait and has been validated and standardized in more than 2000 children at the age of 2–6 years in the UK, the Netherlands, and the U.S. In the standardization sample, split-half reliability was .66 for boys and .80 for girls, test–retest reliability over a 1-year period was .62 for boys and .66 for girls, the correlation between parent and teacher ratings was .37 for boys and .48 for girls, and factor analysis suggested that the items could be used as a one-factor scale (Golombok & Rust, 1993a). In the current sample, internal consistency was high (overall: *α* = 0.94; boys: *α* = 0.90; girls: *α* = 0.92) and confirmatory factor analyses with residual covariance allowed testing the one-factor model showed adequate model fit, overall: *χ* ^*2*^(207) = 527.03, *p* < .001, CFI = .94, RMSEA = .06 [.06, .07]; boys: *χ* ^*2*^(207) = 405.41, *p* < .001; CFI = .91, RMSEA = .07 [.06, .08]; girls: *χ* ^*2*^(207) = 409.87, *p* < .001, CFI = .91, RMSEA = .07 [.06, .08]. The feminine items were reversed scored, and standardized scoring was such that higher total scores represent more male-typical/less female-typical play behavior and lower total scores represent more female-typical/less male-typical play behavior (Golombok & Rust, 1993a). The PSAI contains an activity item assessing fighting. It is likely that this item was interpreted by the parents as a question about playful behavior, rather than aggressive behavior, because it was embedded among other play behavior items in the activity section on the PSAI. Given that scoring with and without the fighting item generated almost identical scores in the current sample (overall: *r* = 0.99; boys: *r* = 0.99; girls: *r* = 0.99), the standard scoring method based on all the items was used.^1^

#### Physical Aggression at Age 13 Years

The RAI (Reinisch & Sanders, 1986) is designed to assess an individual’s tendency toward aggressive behavior. Responses to six descriptions of hypothetical situations involving interpersonal conflicts are assessed. For example, one situation is described as “You’re walking down the street. Some kid is mad at you and comes up and hits you. What do you do?” For each situation, participants were presented with six pairs of forced-choice alternatives (all possible pairwise combinations of four responses—physical aggression, verbal aggression, withdrawal, and non-aggressive coping). Examples of response options include “hit them” (physical aggression), “yell at them” (verbal aggression), “tell an adult” (non-aggressive coping), and “leave them” (withdrawal). There are thus 36 forced-choice questions in total. A score of 1 was given to responses chosen for each pair of forced-choice alternatives, resulting in a possible score range of 0–18 for each of the four responses. Higher scores indicate higher frequency of the responses. Descriptive statistics of RAI scores are shown in Table 2.

**Table 2:** Descriptive statistics of Reinisch Aggression Inventory (RAI) scores within each sex and within each childhood gender-typed play behavior group

	Boys *n* = 199		Girls *n* = 193		Masculine children *n* = 124		Control children *n* = 122		Feminine children *n* = 146	
	*M*	*SD*	*M*	*SD*	*M*	*SD*	*M*	*SD*	*M*	*SD*
Physical aggression	7.01	4.24	3.50	3.59	6.73	4.30	4.93	4.06	4.34	4.21
Verbal aggression	11.57	2.76	10.60	3.40	11.60	2.91	10.91	3.48	10.80	2.94
Non-aggressive coping	9.70	3.43	10.85	3.78	9.57	3.57	10.52	3.83	10.64	3.51
Withdrawal	7.68	3.68	10.95	3.17	8.03	3.54	9.57	3.73	10.12	3.82

The absolute range for the four RAI variables is 0–18

### Control Variables

The ALSPAC data set includes approximately 10,000 variables for each pregnancy. Variables that have been related to aggression in childhood and/or adolescence in previous research were identified (Calkins & Fox, 2002; Côté, Vaillancourt, LeBlanc, Nagin, & Tremblay, 2006; Dionne, Tremblay, Boivin, Laplante, & Perusse, 2003; Loeber & Hay, 1997; Tremblay et al., 2004). These included demographic factors such as maternal age at delivery, maternal education, family income, and presence of sibling(s). Measures of maternal characteristics during prenatal and postnatal periods, such as psychopathology, alcohol consumption, smoking, and quality of parenting, were also considered. Furthermore, child characteristics during early childhood, such as temperament, expressive language, and emotional and behavioral development, were examined.

#### Demographic Background

Four demographic factors were assessed: (1) maternal age at delivery; (2) mother’s highest educational attainment; (3) family income; and (4) presence of siblings(s) (whether or not the child had any siblings; 1 = yes, 0 = no). Both maternal education and family income were categorized into five levels, with 1 being the lowest (maternal education: Certificate of Secondary Education (CSE); family income: <£100 per week) and 5 the highest (maternal education: university degree; family income: >£400 per week).

#### Maternal Characteristics during Prenatal and Postnatal Periods

Psychopathology was measured by the Crown-Crisp Experiential Index (CCEI; Crown & Crisp, 1979), an inventory assessing anxiety, depression, and somatic symptoms, with higher scores indicating higher levels of overall psychopathology. The CCEI was completed by mothers at 18 weeks and 32 weeks of gestation and at 8 weeks, 8 months, and 21 months postnatal. Alcohol consumption was measured by an item inquiring about frequency of drinking alcoholic drinks. The item was scored from 1 (never) to 6 (at least 10 glasses every day). This item was used at 18 weeks of gestation to measure alcohol consumption during the first 3 months of pregnancy and at 8 weeks postnatal to measure alcohol consumption during the last 2 months of pregnancy. Smoking behavior was measured by the mother’s indication of whether or not she had smoked (1 = reported smoking, 0 = no smoking behavior) during the first 3 months of pregnancy (measured at 18 weeks of gestation) and during the last 2 months of pregnancy (measured at 8 weeks postnatal). Quality of parenting was assessed by 10 items on frequency of parenting activities engaged in by the mother at 38 months postnatal. Items addressed various activities, such as bathing, cuddling, and playing. Each item was scored from 1 to 4 indicating: “never,” “rarely,” “sometimes,” and “often.” Higher scores indicate more frequent engagement in parenting activities.

#### Child Characteristics During Early Childhood

Questionnaires were completed by the mothers. For all measures, higher scores indicate higher levels of the traits. Temperamental characteristics, including activity, intensity, persistence, and distractibility, were measured by the Infant Temperament Questionnaire (Carey & McDevitt, 1978) at 6 months of age and the Toddler Temperament Scale (Fullard, McDevitt, & Carey, 1984) at 24 months of age. Activity refers to the child’s physical activity level. Intensity refers to the child’s intensity level of a positive or negative response to a situation. Persistence and distractibility are traits representing the child’s ability to stay focused during tasks. Expressive language, as indicated by the number of words the child can speak, was measured by the MacArthur Communicative Development Inventories (MacArthur CDI; Fenson et al., 1993). The Infant Vocabulary Form was used at 15 months of age, and the Toddler Vocabulary Form was used at 24 months of age. Emotional and behavioral development was measured by the Rutter revised preschool scales (Elander & Rutter, 1996) at age 3.5 years. The scales consist of 43 items in total and assess emotional problems, hyperactivity, conduct problems, and prosocial behavior. Each item was scored from 0 to 2, and response options include: “not true,” “sometimes true,” and “certainly true.”

## Results

### Initial Analyses

Prior to data analyses, continuous variables were first examined for the existence of extreme scores and outliers (*z* = ±3.29) were removed. Continuous variables were then examined for normality. Distributions of values for physical aggression at age 13 years, vocabulary at 8 and 24 months of age, and postnatal maternal psychopathology at 8 weeks, 8 months, and 21 months after the child’s birth were skewed (skewness = ±1). Therefore, a square-root transformation was carried out on these variables. Unless otherwise specified, for these transformed variables, statistical analyses were based on transformed values, whereas descriptive statistics were based on original values.

Magnitudes of gender differences in RAI scores were evaluated by Cohen’s *d* statistic, with positive values indicating higher scores in boys and negative values indicating higher scores in girls. There were large gender differences in physical aggression (*d* = 0.88) and withdrawal (*d* = –0.95). Small-to-medium gender differences were found in verbal aggression (*d* = 0.31) and non-aggressive coping (*d* = –0.32).

Pearson’s correlation analyses were conducted to examine associations between RAI scores and control variables. Correlations between RAI scores for the four responses were also examined. Results are summarized in Table 3. Control variables that significantly correlated with RAI scores were included in subsequent analyses as covariates.

**Table 3:** Correlations between Reinisch Aggression Inventory (RAI) scores for the four responses and between control variables and RAI scores

	Physical aggression	Verbal aggression	Non-aggressive coping	Withdrawal
Reinisch Aggression Inventory *scores*				
Physical aggression (*n* = 392)	–			
Verbal aggression (*n* = 392)	.36***	–		
Non-aggressive coping (*n* = 392)	−.65***	−.64***	–	
Withdrawal (*n* = 392)	−.74***	−.55***	.30***	–
*Socioeconomic background*				
Maternal age at delivery (*n* = 392)	.09	.10*	−.04	−.12*
Maternal education (*n* = 383)	−.00	.01	.07	−.08
Family income (*n* = 304)	−.07	−.01	.11	−.01
Presence of sibling(s) (*n* = 352)	−.02	−.01	.04	−.01
*Maternal characteristics during prenatal and postnatal periods*				
Psychopathology at 18 weeks of gestation (*n* = 363)	.02	.04	−.07	−.02
Psychopathology at 32 weeks of gestation (*n* = 374)	.08	.11*	−.07	−.01
Psychopathology at 8 weeks postnatal (*n* = 380)	.02	.06	−.04	−.05
Psychopathology at 8 months postnatal (*n* = 378)	−.01	.03	−.03	−.01
Psychopathology at 21 months postnatal (*n* = 372)	.03	.05	−.05	−.07
Alcohol consumption during first 3 months of pregnancy (*n* = 389)	.02	.06	−.01	−.04
Alcohol consumption during last 2 months of pregnancy (*n* = 377)	.07	.04	−.08	−.02
Smoking behavior during first 3 months of pregnancy (*n* = 389)	.04	.00	.00	−.04
Smoking behavior during first 2 months of pregnancy (*n* = 380)	.06	−.01	−.02	−.03
Parenting quality at 38 months postnatal (*n* = 376)	.05	.03	−.03	−.07
*Child characteristics in early childhood*				
Activity at age 6 months (*n* = 382)	−.00	.05	−.04	−.02
Activity at age 24 months (*n* = 353)	.13*	.20***	−.17**	−.15**
Intensity at age 6 months (*n* = 381)	.09	.02	−.09	−.05
Intensity at age 24 months (*n* = 338)	−.03	.02	−.01	.01
Persistence at age 6 months (*n* = 382)	−.05	−.06	.03	.03
Persistence at age 24 months (*n* = 348)	−.03	.05	−.01	−.01
Distractibility at age 6 months (*n* = 382)	−.05	−.01	−.01	.07
Distractibility at age 24 moths (*n* = 351)	.00	−.00	−.01	−.03
Vocabulary at age 15 months (*n* = 376)	−.02	−.01	−.02	.06
Vocabulary at age 24 months (*n* = 370)	−.04	−.04	.03	.03
Emotional symptoms at age 3.5 years (*n* = 391*)*	−.12*	−.09	.07	.12*
Hyperactivity at age 3.5 years (*n* = 392)	.13*	.04	−.06	−.11*
Conduct problems at age 3.5 years (*n* = 392)	.12*	.03	−.04	−.11*
Prosocial behavior at age 3.5 years (*n* = 392)	−.17**	−.05	.11*	.13**

* *p* < .05, ** *p* < .01, *** *p* < .001

### Childhood Gender-Typed Play Behavior and Aggression at Age 13 Years

A series of 2 (Sex) × 3 (Childhood Gender-Typed Play Behavior: Masculine, Control, Feminine) analyses of covariance (ANCOVAs) were conducted to examine group differences of sex and childhood gender-typed play behavior in RAI scores at age 13. The *η* ^2^ statistic was used to indicate the percentage of variance accounted for by each of the main effects, interactions, and covariates in the ANCOVA models. Where a significant main effect of childhood gender-typed play behavior was found, simple contrasts were carried out to determine whether masculine children differed from control children, whether feminine children differed from control children, and whether masculine children differed from feminine children. The Cohen’s *d* statistic was used to indicate magnitudes of these pairwise group differences.

#### Physical Aggression

**Fig. 1:**
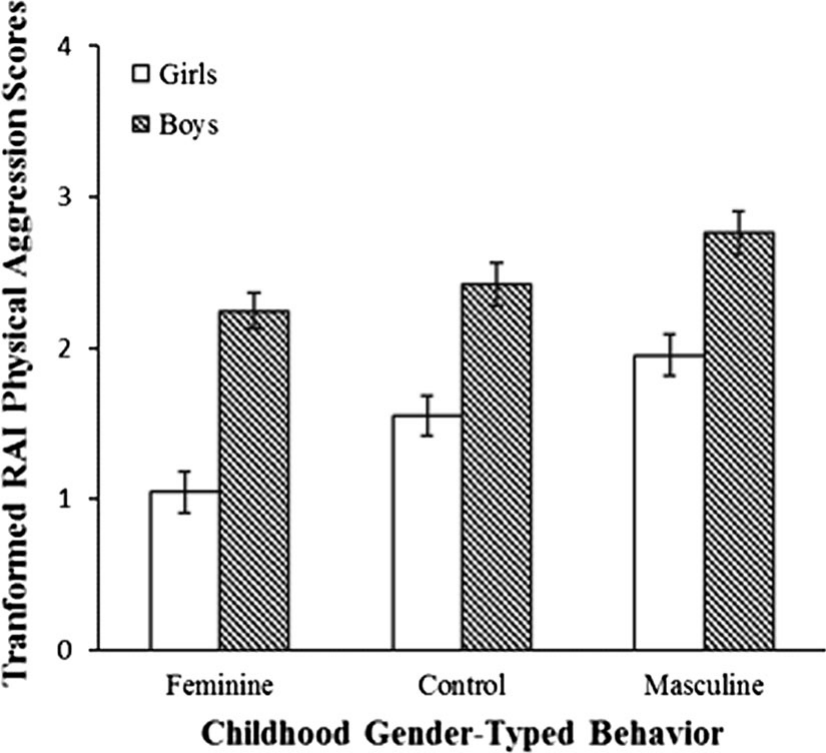
Group differences (M ± 1 SE) in Reinisch Aggression Inventory (RAI) physical aggression scores

There was a significant main effect of sex, *F*(1, 342) = 64.31, *p* < .001, *η* ^2^ = .15, with boys scoring significantly higher than girls, and a significant main effect of childhood gender-typed play behavior, *F*(2, 342) = 8.62, *p* < .001, *η* ^2^ = .04. Simple contrasts revealed that masculine children scored significantly higher than control children, *p* < .05, *d* = 0.38, and feminine children, *p* < .001, *d* = 0.57. Control children also scored significantly higher than feminine children, *p* < .05, *d* = 0.19. The group differences are depicted in Fig. 1. The interaction between sex and childhood gender-typed play behavior was nonsignificant, *F*(2, 342) = 1.40, *p* = .25, *η* ^2^ < .01. Covariates were also nonsignificant: activity at age 24 months: *F*(1, 342) = 0.73, *p* = .40, *η* ^2^ < .01; emotional symptoms at age 3.5 years: *F*(1, 342) = 2.02, *p* = .16, *η* ^2^ < .01; hyperactivity at age 3.5 years: *F*(1, 342) = 0.82, *p* = .37, *η* ^2^ < .01; conduct problems at age 3.5 years: *F*(1, 342) = 0.01, *p* = .94, *η* ^2^ < .01; prosocial behavior at age 3.5 years: *F*(1, 342) = 3.17, *p* = .08, *η* ^2^ < .01.

#### Other Aggression-Related Traits

With regard to verbal aggression, the analysis showed a significant main effect of sex, *F*(1, 332) = 7.14, *p* < .01, *η* ^2^ = .01, such that boys obtained significantly higher scores than girls. However, there was neither a significant main effect of childhood gender-typed play behavior, *F*(2, 332) = 0.30, *p* = .74, *η* ^2^ < .01, nor a significant interaction between sex and childhood gender-typed play behavior, *F*(2, 332) = 1.06, *p* = .35, *η* ^2^ < .01. One covariate, activity at age 24 months, was significant, *F*(1, 332) = 10.99, *p* < .01, *η* ^2^ = .02, indicating that higher activity was associated with higher verbal aggression scores, but the other two covariates were nonsignificant: maternal age at delivery: *F*(1, 332) = 3.60, *p* = .06, *η* ^2^ < .01; maternal psychopathology at 32 weeks of gestation: *F*(1, 332) = 2.07, *p* = .15, *η* ^2^ < .01.

With regard to non-aggressive coping, the analysis showed a significant main effect of sex, *F*(1, 345) = 7.56, *p* < .01, *η* ^2^ = .01, such that girls scored significantly higher than boys. However, there was neither a significant main effect of childhood gender-typed play behavior, *F*(2, 345) = 1.42, *p* = .24, *η* ^2^ < .01, nor a significant interaction between sex and childhood gender-typed play behavior, *F*(2, 345) = 0.13, *p* = .88, *η* ^2^ < .01. One covariate, activity at age 24 months, was significant, *F*(1, 345) = 7.51, *p* < .01, *η* ^2^ = .01, indicating that higher activity was associated with lower non-aggressive coping scores, but the other covariate, prosocial behavior at age 3.5 years, was nonsignificant, *F*(1, 345) = 1.58, *p* = .21, *η* ^2^ < .01.

With regard to withdrawal, the analysis showed a significant main effect of sex, *F*(1, 341) = 77.18, *p* < .001, *η* ^2^ = .19, such that girls had significantly higher scores than boys. There was also a significant main effect of childhood gender-typed play behavior, *F*(2, 341) = 5.04, *p* < .01, *η* ^2^ = .02. Simple contrasts showed that masculine children scored significantly lower than feminine children, *p* < .01, *d* = 0.48. Masculine children also scored lower than control children but the difference was nonsignificant, *p* = .09, *d* = 0.33. Similarly, feminine children scored higher than control children, but the difference was nonsignificant, *p* = .10, *d* = 0.15. The interaction between sex and childhood gender-typed play behavior and effects of covariates was nonsignificant: interaction: *F*(2, 341) = 0.10, *p* = .90, *η* ^2^ < .01; maternal age at delivery: *F*(1, 341) = 2.80, *p* = .10, *η* ^2^ = .01; activity at 24 months: *F*(1, 341) = 2.28, *p* = .13, *η* ^2^ < .01; emotional symptoms at age 3.5 years: *F*(1, 341) = 1.35, *p* = .25, *η* ^2^ < .01; hyperactivity at age 3.5 years: *F*(1, 341) = 0.16, *p* = .69, *η* ^2^ < .01; conduct problems at age 3.5 years: *F*(1, 341) = 0.06, *p* = .81, *η* ^2^ < .01; prosocial behavior at age 3.5 yeas: *F*(1, 341) = 1.30, *p* = .26, *η* ^2^ < .01.

## Discussion

Findings from the present study suggest that childhood gender-typed play behavior predicts physical aggression in early adolescence. Significant group differences in physical aggression were found at age 13 years according to classification as masculine, control, or feminine at age 3.5 years, after controlling for covariates including activity level and traits of emotional and behavioral development in early childhood. As predicted, masculine children showed significantly more physical aggression than control children and feminine children, whereas feminine children showed significantly less physical aggression than control children and masculine children. The interaction between sex and childhood gender-typed play behavior was nonsignificant, suggesting that the association between gender-typed play behavior at age 3.5 and physical aggression at age 13 did not differ between boys and girls.

With respect to verbal aggression, non-aggressive coping, and withdrawal, there was generally a lack of significant differences among masculine, control, and feminine children, suggesting that the relationship between childhood gender-typed play behavior and physical aggression may not extend to all other relevant constructs. Although significant main effects of sex and childhood gender-typed play behavior were found for withdrawal, these effects could reflect the forced-choice nature of the RAI (the four responses depending on one another) and the strong, negative relationship between physical aggression and withdrawal responses (see Table 3).

The current findings may be interpreted to support evolutionary, social, and social-cognitive theories arguing that male-typical play behavior during childhood increases subsequent physical aggression (Archer, 1994; Berkowitz, 1974, 1984; Cline, Croft, & Courrier, 1973; Maccoby, 1998; Pereira & Altmann, 1985). These theoretical perspectives tend to focus on a particular aspect of childhood gender-typed play behavior. For example, the evolutionary perspective focuses on how rough-and-tumble play may help develop fighting skills, whereas the social-cognitive perspective focuses on the subliminal association between aggressive toys and violence. These perspectives thus appear to suggest that different aspects of childhood gender-typed play behavior influence subsequent physical aggression via different mechanisms, although these different aspects of gender-typed play behavior are highly related (Golombok & Rust, 1993a, b). In the present study, instead of focusing on specific aspects, childhood gender-typed play behavior was examined as a single trait using a psychometrically validated measure. While it is possible that these different mechanisms all contribute to the elevated physical aggression observed among masculine children in the present study, further research may usefully explore the specific mechanisms via which gender-typed play behavior may increase subsequent physical aggression.

The present study suggests that childhood gender-typed play behavior predicts subsequent physical aggression in both boys and girls. Some previous research only included boys or a disproportionately small number of girls (Potts et al., 1986; Turner & Goldsmith, 1976). Among prior studies that included both boys and girls, some found a relationship in boys only (Feshbach, 1956; Paquette et al., 2003; Watson & Peng, 1992). In contrast, the present study suggests that the positive association between male-typical play behavior and physical aggression is similar across the sexes.

This inconsistency with prior findings is perhaps due to different sampling methods. Most prior studies were based on small, convenience samples, often including fewer than 50 children in total, and found low prevalence of male-typical play behavior in girls (Feshbach, 1956; Hellendoorn & Harinck, 1997; Ostrov & Keating, 2004; Turner & Goldsmith, 1976; Watson & Peng, 1992). These sampling issues may have limited statistical power and variability in male-typical play behavior among girls. By contrast, the present study included a relatively large sample of randomly selected girls and extremely masculine and feminine girls, which may have provided a more powerful and sensitive measure of childhood gender-typed play behavior in girls.

In addition to the inclusion of a psychometrically constructed measure of childhood gender-typed play behavior and a large, representative sample of boys and girls, the present study benefitted from using a 10-year longitudinal design. Most previous studies focused on concurrent associations during childhood, providing little implications for potential long-term effects beyond childhood (Flanders et al., 2009; Hellendoorn & Harinck, 1997; Ostrov & Keating, 2004; Potts et al., 1986; Turner & Goldsmith, 1976; Watson & Peng, 1992), whereas the present study clearly demonstrated a longitudinal relationship, suggesting that childhood gender-typed play behavior may exert long-term influences on physical aggression across developmental stages.

While it is possible that masculine children already exhibited higher levels of physical aggression during early childhood, it is important to note that the group differences in the present study were found after controlling for childhood hyperactivity, a construct that is strongly correlated with childhood physical aggression (Fontaine et al., 2008; Huijbregts, Séguin, Zoccolillo, Boivin, & Tremblay, 2007; Pingault et al., 2011). The present study also controlled for other behavioral problems as assessed by the Rutter preschool scales, which include items assessing frequency of destroying objects and bullying other children, behaviors that have been used as indicators of childhood physical aggression in previous research (Alink et al., 2006; Tremblay et al., 2004). Therefore, it is unlikely that the positive association observed in the present study is attributable to elevated childhood physical aggression among masculine children.

### Conclusion

Using a 10-year longitudinal design, a large, representative sample of boys and girls, and a psychometrically validated measure of childhood gender-typed play behavior, the present study found that gender-typed play behavior in early childhood predicts physical aggression in early adolescence. Boys and girls who were more masculine as young children exhibited more physical aggression as adolescents. While the current findings may be explained by evolutionary, social, or social-cognitive perspectives, which appear to suggest that different aspects of childhood male-typical play behavior increase subsequent physical aggression via different mechanisms, further research specifically designed to identify the specific mechanisms involved in the association between childhood gender-typed play behavior and later physical aggression would be useful.

## Footnotes

1 Analyses based on scoring without the fighting item did not change the results or conclusions of the current study.
